# Prevalence and detection of *Brucella* infection in people with fever of unknown origin in Inner Mongolia, China

**DOI:** 10.3389/fcimb.2026.1838461

**Published:** 2026-06-17

**Authors:** Tianlong Zhang, Jing Li, Yan Hai, Zhen Wang, Mingmei Wang, Yingdi Wang, Ying Zhang, AiPing Qin, Jie Liu

**Affiliations:** 1 School of Public Health, Qingdao University, Qingdao, China; 2 General Center for Disease Control and Prevention of Inner Mongolia Autonomous Region, Hohhot, Inner Mongolia Autonomous Region, China; 3 Center for Disease Control and Prevention of Xilingol League, Xilinhaote, Inner Mongolia Autonomous Region, China; 4 National Institute for Communicable Disease Control and Prevention, Chinese Center for Disease Control and Prevention, Beijing, China

**Keywords:** *Brucella*, ELISA - enzyme-linked immunosorbent assay, fever of unknown origin, pathogen load, quantitative polymerase chain reaction, zoonosis

## Abstract

**Background:**

Brucellosis is a highly endemic zoonosis in Inner Mongolia, yet its contribution to undifferentiated febrile illnesses often remains underestimated due to non-specific clinical presentations and diagnostic limitations. This study aimed to determine the prevalence and diagnostic gaps of *Brucella* infection among patients presenting with fever of unknown origin (FUO) in Inner Mongolia.

**Methods:**

A cross-sectional surveillance study was conducted in 2021. A total of 335 febrile patients were enrolled, with 266 meeting the criteria for FUO. Venous blood samples were collected and tested using quantitative real-time PCR (qPCR) followed by digital PCR (dPCR) for absolute quantification of bacterial loads. A subset of serum samples was tested with ELISA for total antibody.

**Results:**

Of the 335 febrile patients, 228 (68.0%) were farmers or herders and 247 (73.7%) had livestock contact. *Brucella* was detected in 52 (15.5%), with 40 (15.0%) in 266 FUO patients. A substantial portion of *Brucella* detection was identified in people (22/107, 18.7%) with professions other than farmer or herder. The history of direct contact with livestock had no association with *Brucella* detection. The most common clinical symptom among positive patients was muscle, joint, or musculoskeletal pain (84.6%, 44/52), but it lacked the specificity for *Brucella* detection. dPCR determined the bacterial loads ranging from 1.7 × 10^2^ to 1.3 × 10^5^ copies/mL of blood, demonstrating a declining trend along with the interval between fever onset and blood draw in FUO patients. ELISA identified three additional cases of *Brucella* infection in the subset of 236 FUO patients.

**Conclusion:**

This surveillance confirms that *Brucella* is a significant causative agent of FUO in the surveillance area, accounting for 15.0% of cases. The sole reliance on serological tests in clinical settings may lead to missed diagnoses. The integration of sensitive qPCR methods is crucial for improving diagnostic accuracy and timely case detection. These findings underscore the need for enhanced clinical awareness and the implementation of routine *Brucella* screening in FUO algorithms across healthcare facilities in Inner Mongolia to reduce diagnostic delays and prevent chronic complications.

## 1 Introduction

*Brucella* spp. are highly pathogenic bacteria that can enter the human body through compromised skin, mucous membranes, and respiratory or digestive tracts, leading to brucellosis, a zoonotic infectious disease affecting humans, livestock, and wildlife (García-Méndez et al., 2019). Human *Brucella* infection primarily occurs through contact with infected animals, particularly livestock such as cattle, sheep, and pigs, as well as their by-products (e.g., raw milk, cheese) (Schelling et al., 2003). The disease in animals usually causes reproductive failure, including abortion, stillbirth, and infertility. From a One Health standpoint, effective diagnosis requires methods that mantle species and sample types across transmission routes while informing surveillance, disease control, and outbreak response (Patel, 2026).

In China, *Brucella* infection predominantly clusters in the western and northern regions, particularly in the interface between agriculture and animal husbandry (Zhang et al., 2011). Among these areas, the Inner Mongolia Autonomous Region faces a particularly challenging epidemic prevention and control scenario, owing to its substantial reliance on animal husbandry and the intimate interaction between herders’ daily lives and livestock (Cao et al., 2025). Inner Mongolia has a historical prevalence of brucellosis, which was effectively controlled in the 1980s (Zhang et al., 2024). However, the rapid expansion of animal husbandry and breeding practices has led to a resurgence in the incidence of brucellosis (Zheng et al., 2018). Since 2017, there has been a notable increase in the prevalence of brucellosis in Inner Mongolia (data sourced from the National Infectious Disease Report Information Network), with a total of 210,177 accumulated cases of human brucellosis reported from 2001 to 2021, reaching to history high value of 21,910 in 2021 (Liu et al., 2025a). *Brucella melitensis* has been identified as the predominant species in the region, accounting for >95% of the brucellosis cases, followed by *Brucella abortus* and occasionally *Brucella suis* (Chen et al., 2013; Liu et al., 2020).

*Brucella* pathogenesis utilizes a unique strategy, defined by intracellular replication within ER-derived compartments, T4SS-dependent modulation of host trafficking, and chronic persistence despite a functional immune system, often leading to relapsing fever, osteoarticular complications, and difficulty in eradication. *Brucella* infection has an incubation period typically ranging from 1 to 4 weeks, with clinical manifestations encompassing recurrent fever, profuse sweating, joint pain, hepatosplenomegaly, and, in severe cases, endocarditis, meningitis, or chronic fatigue syndrome (Lim and Rickman, 2004; Abbas et al., 2025). However, differential diagnosis is required, particularly to be differentiated from typhoid fever, malaria, tuberculosis, lymphoma, dengue fever, leptospirosis, rheumatic disease, etc (Araj, 2010). Additionally, diagnosis of brucellosis may rely on different methods during the disease progression (Di Bonaventura et al., 2021). Without specific laboratory confirmation, misdiagnosis is common, leading to delayed treatment (Soares et al., 2023). This delay can allow the infection to become chronic, resulting in debilitating complications such as arthritis, spondylitis, and endocarditis, which cause long-term suffering and economic loss for families (Hanot Mambres et al., 2017).

Bacterial culture is the gold standard method for *Brucella* detection, which confirms active infection (Xu et al., 2023). However, it is time-consuming due to slow growth and often has low yield, especially in chronic cases where bacteria are intermittent in the blood. More importantly, *Brucella* culture required Biosafety Level 3 facilities, which is not always readily available (Klietmann and Ruoff, 2001). Due to its undulant fever pattern, slow-growing nature and intracellular sequestration, fever of unknown origin (FUO) is a common clinical presentation of brucellosis (Wu et al., 2022). When the disease is systemic, antibody titers are usually detectable while culture is negative. Thus, enzyme-linked immunosorbent assay (ELISA), a serological test, is the most commonly used method, which is simple and rapid for screening the immune response to infection, but its performance can vary between commercial kits (Fadeel et al., 2011). PCR, on the other hand, is highly sensitive, specific, and useful for the detection of the pathogen per se in FUO cases, especially when the patient has started antibiotic treatment (Wright et al., 2022). The performance of PCR depends on the choice of target sequence.

## 2 Materials and methods

### 2.1 Participants and sample collection

Patients with febrile illness but no etiology identified were enrolled from 10 of the 12 districts of the Inner Mongolia Autonomous Region between March and December 2021. FUO was defined according to the guidelines of the China Centers for Disease Control and Prevention (CDC) as fever >38.3°C (101°F) lasting for more than 3 weeks with no confirmed diagnosis after a thorough medical history investigation, physical examination, and routine laboratory tests (Expert Consensus Group, 2017). Blood samples were collected upon enrollment. Whole blood and serum prepared were stored at −80°C until testing. Sociodemographic information and clinical symptoms were recorded, including age, gender, occupation, permanent residence, onset time of fever, duration of fever, and accompanying symptoms (such as headache, fatigue, muscle soreness, chills). All the Institutional Review Boards approved this work. Written informed consent was obtained from each participant.

### 2.2 Nucleic acid extraction

Total nucleic acid was extracted from 200 μL of whole blood using the Biospin Virus DNA/RNA Extraction Kit (BIOER, Zhejiang, China) following the manufacturer’s instructions. Briefly, the samples were lysed, and the lysate went through a specialized polymer membrane followed by wash and elution steps. Phocine herpesvirus (PhHV) and MS2 bacteriophage were spiked into each sample to monitor the extraction/amplification efficiency (Marks et al., 2021). Each extraction batch included a negative control sample to control for potential laboratory contamination.

### 2.3 qPCR for *Brucella* detection

qPCR assay targeting *IS711* was performed for *Brucella* detection, which was extensively validated previously (Becker and Tuon, 2021). The primer and probe sequences are shown in **Supplementary Table 1**. The 10-μL qPCR reaction contained 2 μL of HiScript III U+ One Step qRT-PCR Probe 5 × Master Mix (Vazyme, Nanjing, China), 9 pmol of primers, 2.5 pmol of probe, and 2 μL of nucleic acid extract and was carried out with a QuantStudio7 Real-time PCR instrument. The cycling conditions included reverse transcription at 55°for 15 min, initial denaturation at 95°C for 10 s, then 40 cycles of 95°C for 30 s and 60°C for 30 s. A positive result (quantification cycle, Cq < 40) is valid only when the corresponding extraction blank is negative.

### 2.4 PCR speciation of *Brucella*

*Brucella* qPCR positives were further speciated with nested PCR for *B. melitensis* and *B. abortus*, with primers shown in **Supplementary Table 1**. The first-round nested PCR was performed in a total volume of 25 μL, containing 12.5 μL of 2×μTaqPlus (Vazyme, Nanjing, China), 10 pmol each of the forward and reverse primers, 2 μL of DNA template, and up to 25 µL of nuclease-free water. The amplification conditions included pre-denaturation at 95 °C for 3 min, 30 cycles of denaturation at 95 °C for 15 s, annealing at 60 °C for 20 s, extension at 72 °C for 60 s, and final extension at 72 °C for 7 min. Two microliters of the first-round product was used for the second-round PCR with the same cycling conditions as the first round.

### 2.5 Digital PCR for absolute quantification

Digital PCR (dPCR) was performed using the same qPCR assay targeting *IS711* with QIAcuity One (QIAGEN, Hilden, Germany) using the 8.5K 24-well Nanoplate and QIAcuity Probe PCR Kit. The 12-μL reaction contained 3 μL of 4× Master Mix, 10.8 pmol of primers, 3 pmol of probe, 1 μL of the restriction enzyme *Xba*I, and 7.5 μL of nucleic acid extract. The cycling conditions were the same as qPCR. The analysis was done using QIAcuity Software Suite.

### 2.6 Serological test

The serological test was performed using 200 μL of serum using the Human Brucellosis Antibody Enzyme-Linked Immunosorbent Assay (ELISA) kit (JINGMEI, Jiangsu, China). The absorbance (OD value) was measured by a microplate reader (DROIDE, Shanghai, China) at 450 nm wavelength.

### 2.7 Statistical analysis

Statistical analysis was conducted using GraphPad Prism 10.0 and SPSS 21.0 software. The Pearson *χ*^2^ test was employed, with a significance level of *α* = 0.05. The two-tailed *P*-value was calculated, and a *P*-value <0.05 was considered statistically significant.

## 3 Results

### 3.1 Demographics of febrile cases

Of the 335 patients with febrile illness from 10 leagues/cities in the Inner Mongolia Autonomous Region (**Supplementary Figure 1**), the median age was 55 (IQR 47–62) years, and 57.3% (192/335) were women, as shown in **Table 1**. About two-thirds (225/335, 67.2%) of the participants were from the central region, followed by the western region (86, 25.7%). The majority (228/335, 68.0%) of the participants were farmers or herders, followed by those with no fixed profession (35/335, 10.5%) and those retired or unemployed individuals (22/335, 6.6%). There were 247 (73.7%) participants who had a history of contact with livestock, mostly sheep (171/247, 73.7%) and cattle (26/247, 11.2%), or both (35/247, 15.1%). Additionally, the predominant clinical symptom was muscle, joint, or musculoskeletal pains (218/335, 65.1%) (**Table 1**). Only 24 (7.2%) patients did not have any accompanying symptom. Based on the case definition, 79.4% (266/335) of the patients met the criteria for FUO (**Table 1**). No difference in demographics was observed between FUO and those with fever within 3 weeks.

**Table 1:** Clinical and demographic features of 335 fever cases in Inner Mongolia.

Clinical and demographic features	Total, n (%)	Patients with fever <3 weeks	Patients with FUO	P-value
Gender				
Male	192 (57.3%)	41 (59.4%)	151 (56.8%)	0.691
Female	143 (42.7%)	28 (40.6%)	115 (43.2%)	
Age				
0–19	4 (1.2%)	0 (0)	4 (1.5%)	0.005
20–39	43 (12.9%)	11 (15.9%)	32 (12.0%)	
40–59	176 (52.5%)	33 (47.8%)	143 (53.8%)	
60–79	112 (33.4%)	25 (36.2%)	87 (32.7%)	
Profession				
Farmer	187 (55.8%)	42 (60.9%)	145 (54.5%)	0.503
Herder	41 (12.2%)	9 (13.0%)	32 (12.0%)	
Others	107 (32.0%)	18 (26.1%)	89 (33.5%)	
Livestock contact history				
Yes	247 (73.7%)	51 (73.9%)	196 (73.7%)	1
No	88 (26.3%)	18 (26.1%)	70 (26.3%)	
Animal types				
Sheep	171 (73.7%)	40 (78.4%)	131 (66.8%)	0.49
Cattle	26 (11.2%)	6 (11.8%)	20 (10.2%)	
Mixed	35 (15.1%)	5 (9.8%)	30 (15.3%)	
Region (reported population in million in 2021)				
Western Baotou (2.7)	61 (18.2%)	11 (15.9%)	50 (18.8%)	0.846
Ordos (2.2)	15 (4.4%)	4 (5.8%)	11 (4.1%)	
Bayan Nur (1.5)	10 (3.0%)	1 (1.4%)	9 (3.4%)	
Central Hohhot (3.5)	144 (43.0%)	28 (40.6%)	116 (43.6%)	
Ulanqab (1.7)	75 (22.4%)	19 (27.5%)	56 (21.1%)	
Xilingol League (1.1)	6 (1.8%)	0 (0)	6 (2.3%)	
Eastern Tongliao (2.9)	8 (2.4%)	2 (2.9%)	6 (2.3%)	
Hinggan League (1.4)	6 (1.8%)	2 (2.9%)	4 (1.5%)	
Chifeng (4.0)	2 (0.6%)	0 (0)	2 (0.8%)	
Hulunbuir (2.2)	1 (0.3%)	0 (0)	1 (0.4%)	
Others	7 (2.1%)	2 (2.9%)	5 (1.9%)	
Clinical symptoms				
Muscle, joint, or musculoskeletal pain	282 (84.2%)	53 (59.6%)	229 (71.3%)	0.01
Headache	21 (6.3%)	10 (11.2%)	11 (3.4%)	
Sweating	19 (5.7%)	4 (4.5%)	15 (4.7%)	
Fatigue	14 (4.2%)	1 (1.1%)	13 (4.0%)	
Shiver	5 (1.5%)	1 (1.1%)	4 (1.2%)	
Lymphadenectasis	3 (0.9%)	1 (1.1%)	2 (0.6%)	
Diarrhea, abdominal pain	3 (0.9%)	0 (0)	3 (0.9%)	
Others	39 (11.6%)	8 (9.0%)	31 (9.7%)	
None of the above	24 (7.2%)	11 (12.4%)	13 (4.0%)	

### 3.2 *Brucella* detection with PCR

qPCR targeting *IS711* identified 52 (15.5%) *Brucella* positives among 335 febrile cases. Further speciation identified six cases of *B. melitensis* and three *B. abortus*, which exclusively had lower *IS711* Cq values (30.4 ± 4.1 versus 34.8 ± 2.2 for those unspeciated, *P* < 0.05). The male patients had higher *Brucella* detection than female patients (37/192, 19.3% versus 15/143, 10.5%, *P* = 0.033, **Table 2**). There was no difference in *Brucella* detection with or without livestock contact history. No association with any clinical symptom was observed. The detection in the western region was the highest (18/86, 20.9%), versus 14.7% (33/225) and 5.9% (1/17) in the central and eastern regions, respectively (*P* < 0.05).

**Table 2:** Detection of *Brucella* with qPCR in patients with fever or FUO in Inner Mongolia.

Clinical and demographic features	Overall detection of Brucella	Patients with fever <3 weeks	Patients with FUO
Gender			
Male	37 (19.3%)	9 (22.0%)	28 (18.5%)
Female	15 (10.5%)	3 (10.7%)	12 (10.4%)
Age			
0–19	0(0)	0 (0)	0 (0)
20–39	6 (14.0%)	2 (18.2%)	4 (12.5%)
40–59	27 (15.3%)	5 (15.2%)	22 (15.4%)
60–79	19 (17.0%)	5 (20.0%)	14 (16.7%)
Profession			
Farmer	24 (12.8%)	8 (19.0%)	16 (11.0%)
Herder	8 (19.5%)	0 (0)	8 (25.0%)
Others	22 (18.7%)	4 (22.2%)	16 (18.0%)
Livestock contact history			
Yes	41 (16.6%)	9 (17.6%)	32 (16.3%)
No	11 (12.5%)	3 (16.7%)	8 (11.4%)
Animal types			
Sheep	33 (21.1%)	7 (17.5%)	26 (19.9%)
Cattle	4 (15.4%)	2 (33.3%)	2 (10.0%)
Mixed	3 (8.6%)	1 (20.0%)	2 (6.7%)
Region (reported population in million in 2021)			
Western Baotou (2.7)	12 (19.7%)	1 (9.1%)	11 (22.0%)
Ordos (2.2)	3 (20.0%)	1 (25.0%)	2 (18.2%)
Bayan Nur (1.5)	3 (30.0%)	0 (0)	3 (33.3%)
Central Hohhot (3.5)	18 (12.5%)	6 (21.4%)	12 (10.3%)
Ulanqab (1.7)	15 (20.0%)	4 (21.1%)	11 (19.6%)
Xilingol League (1.1)	0 (0)	0 (0)	0 (0)
Eastern Tongliao (2.9)	0 (0)	0 (0)	0 (0)
Hinggan League (1.4)	1 (16.7%)	0 (0)	1 (25.0%)
Chifeng (4.0)	0 (0)	0 (0)	0 (0)
Hulunbuir (2.2)	0 (0)	0 (0)	0 (0)
Others	0 (0)	0 (0)	0 (0)
Clinical symptoms			
Muscle, joint or musculoskeletal pain	44 (15.6%)	9 (17.0%)	35 (15.3%)
Headache	3 (14.3%)	2 (20.0%)	1 (9.1%)
Sweating	4 (21.1%)	1 (25.0%)	3 (20.0%)
Fatigue	1 (7.1%)	0 (0.0%)	1 (7.7%)
Shiver	1 (20%)	1 (100.0%)	0 (0.0%)
Lymphadenectasis	0 (0)	0 (0.0%)	0 (0.0%)
Diarrhea, abdominal pain	1 (33.3%)	0 (0.0%)	1 (33.3%)
Others	10 (25.6%)	2 (25.0%)	8 (25.8%)
None of the above	4 (16.7%)	2 (18.2%)	2 (15.4%)

Among the 266 FUO patients, 15.0% (40) were positive for *Brucella*, with no difference from those with fever within 3 weeks (12/69, 17.4%). Compared to farmers with FUO (16/129, 11.0%), herders with FUO had higher *Brucella* detection (8/32, 25.0%, *P* = 0.047). A higher detection rate was observed in male FUO patients, but it lack statistical significance (28/151, 18.5% versus 12/115, 10.4%, *P* = 0.083). Animal contact history had no association with the detection of *Brucella* in FUO patients.

**Figure 1:**
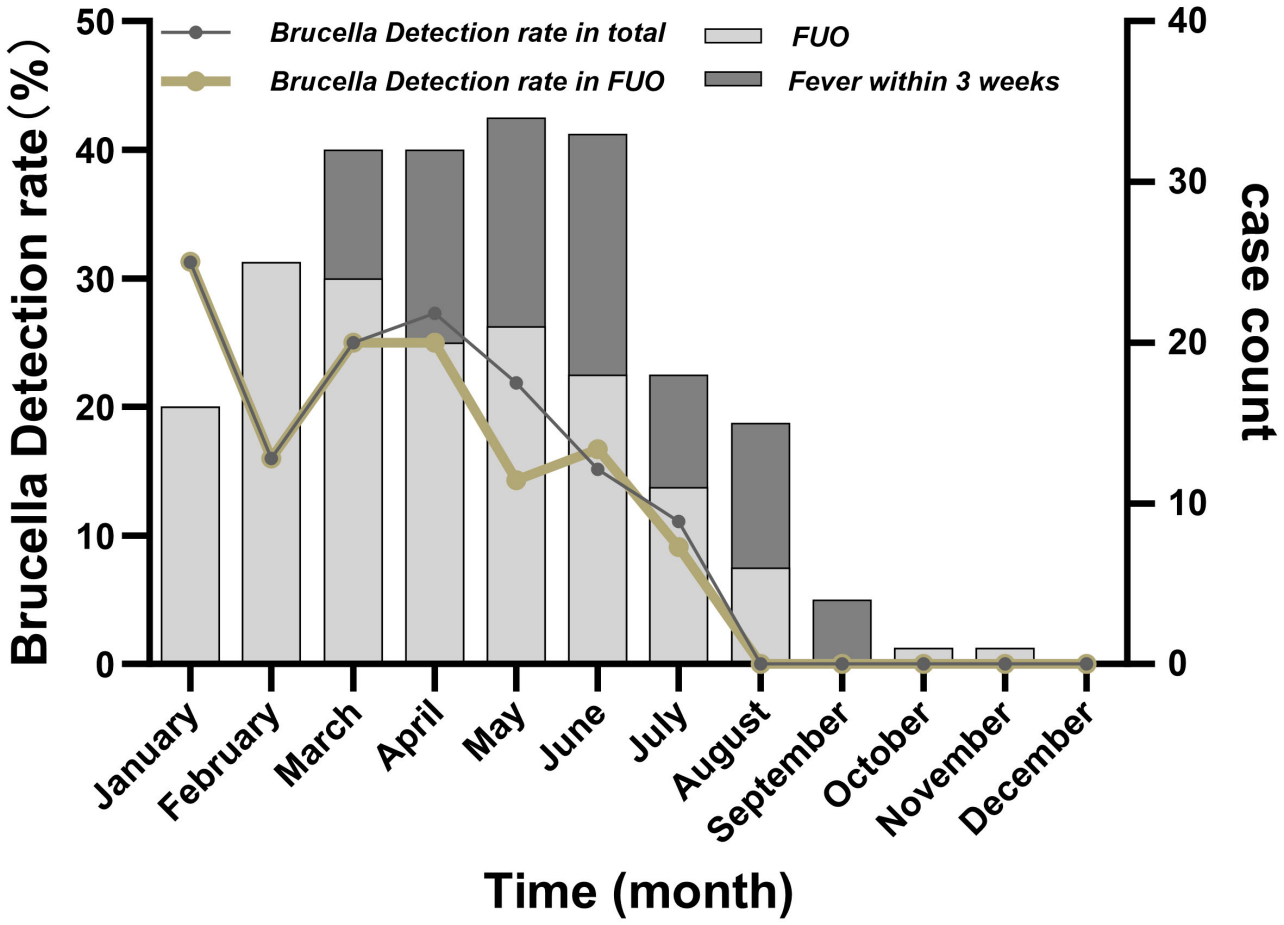
Temporal distribution of *Brucella* detection in patients with fever or FUO in Inner Mongolia, 2021.

Most of the febrile patients had a fever onset time between January and August, while the detection of *Brucella* showed an obvious increase from February to April and then gradually declined afterwards (**Figure 1**).

### 3.3 *Brucella* quantification with dPCR

Absolute *Brucella* loads were assessed in 40 available samples using dPCR. Excluding those with <3 positive wells (*n* = 16), 24 samples were further quantified for copy numbers. A negative correlation between Cq values and copy numbers was observed (*R*^2^ = 0.455, *P* = 0.0003, **Supplementary Figure 2**) as expected. The copy numbers identified by dPCR ranged from 1.7 × 10^2^ to 1.3 × 10^5^ copies/mL of blood. The distribution of *Brucella* loads is shown in **Supplementary Figure 3** for genders, age groups, professions, contact history, and clinical symptoms.

**Figure 2:**
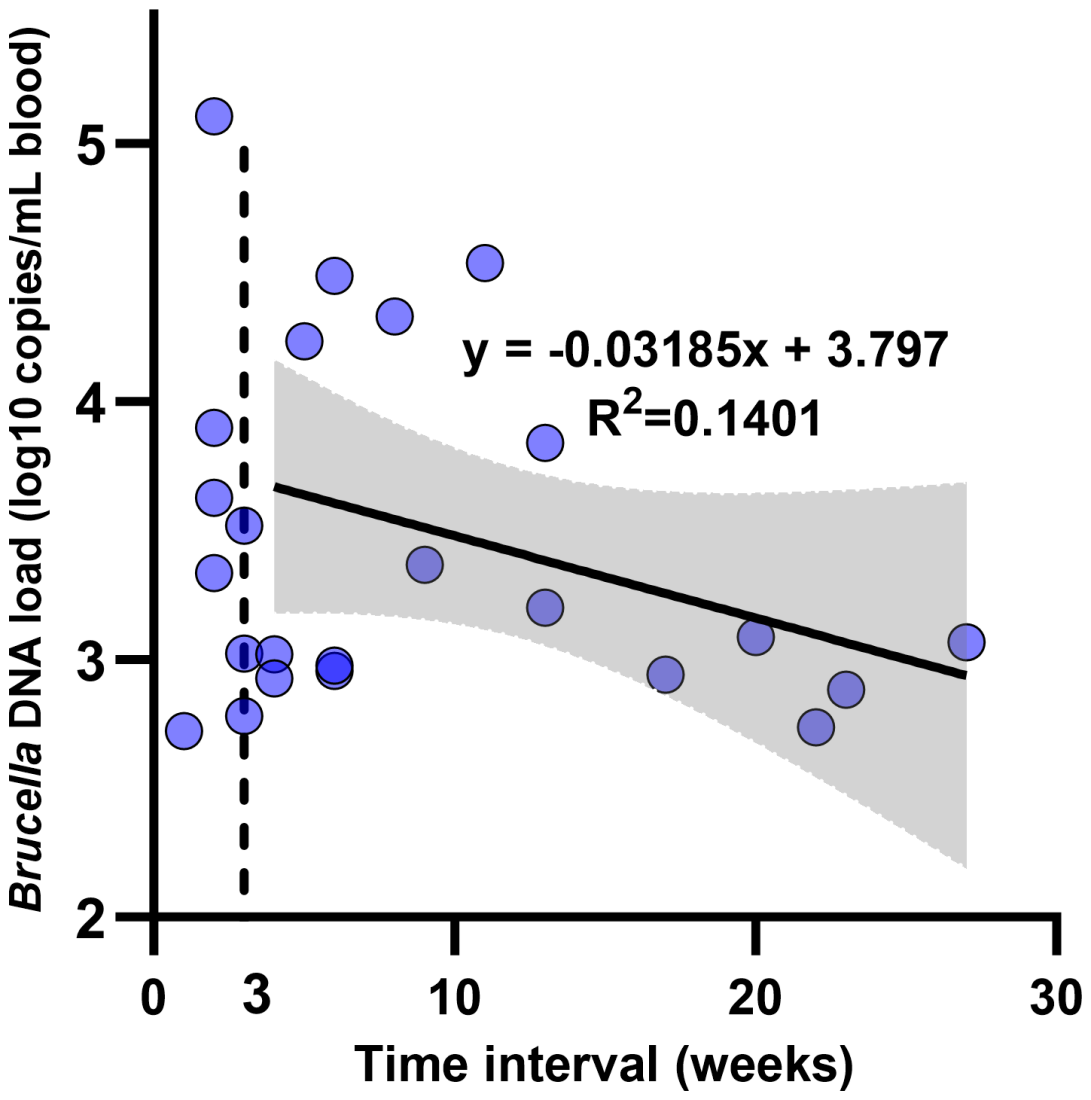
*Brucella* bacterial loads in febrile patients by time interval between symptom onset and blood draw. *Brucella* bacterial loads are shown as log10 copies per mL of blood. The dashed line indicates the 3-week mark for FUO.

The relationship between the interval of fever onset and blood draw with *Brucella* loads was further explored. The averaged interval between fever onset and blood draw was 68.3 ± 69.7 days. The patients with *Brucella* detected had shorter interval than those with negative results (58.9 ± 53.8 versus 91.9 ± 70.3 days, *P* < 0.05). There was no difference in *Brucella* loads between FUO patients and those with fever within 3 weeks, while a declining trend was indicated for FUO patients with the elongation of interval for blood draw in **Figure 2**.

### 3.4 Serological tests with ELISA

A subset of 236 participants with sufficient serum samples available were tested with ELISA for total antibody against *Brucella* (IgG+IgM). According to the positivity definition of the ELISA kit (OD ≥ 0.15), only 6 (2.5%) cases were positive, exclusively with FUO. Only one was detected as positive for *Brucella* by qPCR.

## 4 Discussion

The early and accurate detection of *Brucella* in patients presenting with fevers of unknown origin in Inner Mongolia is of critical public health importance (Liu et al., 2025b). Given that the region’s economy and culture are deeply intertwined with livestock husbandry, particularly sheep and cattle, the population faces a high occupational risk of exposure to brucellosis (Liu et al., 2024). In the current study, we investigated *Brucella* detection in febrile patients, mostly FUO cases, from most of the regions in Inner Mongolia using qPCR followed by dPCR for accurate quantification and ELISA. *Brucella* was detected in 15.0% of the FUO patients, with a higher risk in men and herders. Additionally, several cases were identified by ELISA. Similar *Brucella* detection rates have been reported in FUO patients. A serological investigation of individuals with unexplained fever in Xinjiang’s farming-pastoral areas in 2023 reported a positive rate of 18.5% (58/313) using a dipstick assay for *Brucella* antibody detection (Guo et al., 2023).

Despite decades of control efforts, brucellosis has exhibited remarkable persistence in Inner Mongolia, with incidence rates rising from 18.27/100,000 in 2004 to 68.34/100,000 in 2023 (Wang M. et al., 2025), which is believed to be attributable to a complex, self-reinforcing system of ecological, economic, behavioral, and institutional determinants. The government has shifted toward a “four-pronged” comprehensive strategy, including vaccination (mass immunization of livestocks), culling (strict removal of infected animals), disinfection (environmental control of contaminated areas), and health education (public awareness campaigns). The surrounding Asian countries share key similarities in brucellosis burden, including predominant *B. melitensis* lineage (Ali et al., 2024), persistent challenges of nomadic lifestyles and livestock being the only source of livelihood, and underestimated incidence. Therefore, the fight for brucellosis represents a regional challenge requiring cooperation and information sharing on epidemiology.

The diagnosis of brucellosis in Inner Mongolia presents a unique set of challenges due to the region’s high endemicity, the non-specific nature of the disease’s clinical presentation, and the limitations of current diagnostic tools. While a relatively complete diagnostic system has been established in China (Liu and Jiang, 2021), there is still no single method that is simultaneously simple, rapid, highly sensitive, and highly specific. This reality leads to significant issues with misdiagnosis and delayed diagnosis, which in turn increase the risk of chronicity, complications, and economic burden. The clinical diagnosis of brucellosis in the region typically relies on a combination of epidemiological history, clinical manifestations, and laboratory testing (Shi et al., 2024). The initial step involves identifying patients with risk factors, such as occupational exposure (herders, veterinarians, slaughterhouse workers) or a history of consuming unpasteurized dairy products. In a large-scale study of 14,506 cases conducted in Tongliao City (Zhai et al., 2021), Inner Mongolia, on factors associated with diagnostic delays in human brucellosis, several risk factors for longer delays were identified, including older age and patients who were not herders or living in non-pastoral areas, indicating that patients outside the classic high-risk groups may be overlooked by clinicians. In the current study, livestock contact history had no association with *Brucella* detection. Additionally, substantial FUO patients were non-herders, with similar *Brucella* detection, particularly among people with other professions. Because its clinical manifestations are diverse and lack specificity, *Brucella* infection is frequently misdiagnosed as upper respiratory tract infection, lumbar disc herniation, or arthritis (Yan et al., 2021). Focal forms of the disease present an even greater diagnostic challenge. A rare manifestation like sternoclavicular arthritis is particularly prone to misdiagnosis, underscoring the need for careful clinical differentiation (Hasanjani Roushan et al., 2016). Our study confirmed that *Brucella* detection showed no association with any of the clinical symptoms.

Serology remains the most commonly used laboratory method for diagnosis and follow-up monitoring in the region due to its simplicity and low cost (Sha et al., 2025). Several tests are available, including the Rose Bengal test (RBT), standard tube agglutination test (SAT), and ELISA. Serological tests such as RBT may yield false positive results due to the lack of specificity, while they can also be falsely negative in the first 1 to 2 weeks of illness before detectable antibodies are produced (Loubet et al., 2024). Meanwhile, serology may also be negative or inconclusive in chronic cases or focal forms like neurobrucellosis (Yuan et al., 2025). As IgG antibodies can persist for years after a cured infection, it can be challenging to distinguish past from current infection using serological tests (Franco et al., 2007). Since the diagnosis of brucellosis in Inner Mongolia has relied heavily on serological screening, diagnostic delays and frequent misdiagnosis may be the ultimate causes for *Brucella* being the main etiology of FUO patients in the current study. To address such issues, a multifaceted approach is essential. This includes maintaining a high index of suspicion in patients with compatible symptoms, even in the absence of classic risk factors (Makashir et al., 2026). It also necessitates the strategic use of complementary tests, such as employing qPCR for early and seronegative cases and relying on expert consensus guidelines for diagnosing complex focal forms of the disease (Lavigne et al., 2025). The goal is to move toward a more integrated diagnostic algorithm that can reduce delays and improve patient outcomes. Integrating PCR into the brucellosis diagnostic workflow may serve as a strategic complement that addresses the inherent limitations of antibody-based detection. For acute early infection before seroconversion, direct detection of bacterial DNA enables diagnosis. On the other hand, for chronic or focal infection, antibody titers may decline or disappear after prolonged illness, while PCR can detect *Brucella* DNA in deep tissues where bacteria persist despite negative serology.

qPCR has been widely utilized for *Brucella* detection because of its high sensitivity and specificity (Wang X. et al., 2025). To achieve superior sensitivity, the choice of target gene is a factor that is not negligible. Screening for *Brucella* was conducted using qPCR targeting the *bcsp31* gene, yielding <1% detection (Zhang et al., 2022). *bcsp31* is a single-copy gene encoding a conserved protein. In this study, to improve the detection of *Brucella*, we used *IS711*, a multicopy insertion sequence (Hinić et al., 2008), yielding an overall of 15.5% *Brucella* detection. Based on the genome sequence analysis, each *Brucella* genome contains 7 to over 30 copies of *IS711*, making it ideal for samples with low bacterial loads such as bloodstream infections, especially during the acute early phase or late stage of shedding.

Absolute quantification using dPCR offers the opportunity for understanding disease progression, monitoring treatment response, and predicting outcomes in brucellosis. Here, we explored the relationship between bacterial loads and the interval between fever onset and blood draw, which revealed a declining trend over months, suggesting a detection window and limit of detection. Worth noting is that the correlation was admittedly weak, which could be affected by the limited sample size in the current study or individual patient status, such as immune response and prior partial treatment. Nonetheless, for patients with chronic brucellosis that is difficult to diagnose and manage, dPCR may be utilized to confirm infection, quantify the residual bacterial burden, and monitor the effectiveness of extended treatment regimens (Liu et al., 2023).

Distinguishability of active infections is a crucial question in molecular diagnostics (Almas et al., 2023). *Brucella* is a facultative intracellular bacterium. Its primary survival strategy is to hide inside host cells (macrophages, dendritic cells, placental trophoblasts) that may enter a dormant or quiescent state (Xiao et al., 2022). Such asymptomatic carriers still harbor the potential for reactivation, especially when they become immunocompromised. Furthermore, when a chronic *Brucella* infection is established that lasts for years, its DNA footprint is even more enduring, often persisting for months to years after clinical cure (Vrioni et al., 2008). This makes PCR an exquisitely sensitive tool for detecting past exposure to the organism, such as this study, where the etiology was identified for FUO with a *Brucella* detection window ranging from weeks to months, but it is an insufficient standalone test for diagnosing active disease. Ultimately, it is essential to understand DNAemia to avoid misdiagnosis and unnecessary treatment (Park et al., 2024).

Either serology or PCR screens for targeted pathogens, limiting detection to those identified based on prior knowledge. Metagenomic sequencing, on the other hand, provides unbiased detection and comprehensive pathogen coverage, which has recently been widely adopted for clinical diagnosis and pathogen surveillance (Torres Montaguth et al., 2026; Wolf et al., 2026). This technology has been particularly powerful in identifying rare or difficult-to-culture pathogens that are often missed by conventional methods, becoming a promising diagnostic tool for FUO (Marra et al., 2024, Gao et al., 2025). Plasma has been strongly preferred for metagenomic sequencing because of its relatively high microbial DNA recovery, low host background, and effective contamination control. Due to the availability of the samples, we explored to implement metagenomic sequencing on serum samples in the current study with the intention to identify additional etiological agents of FUO. However, most of the samples did not pass the quality control, and the limited sequencing data acquired did not yield reliable analysis (data not shown). In future studies, conventional methods, PCR-based molecular diagnostics, and metagenomic sequencing may be incorporated into study design with coordinated sample preparation and workflow for comprehensive evaluation and optimization of diagnostic algorithm. This study has a few limitations. First, the enrollment of febrile patients in the eastern region was underrepresented although participants were from 10 of the 12 districts of Inner Mongolia, except two leagues with the least populations (Wuhai and Alashan League), such as Chifeng and Tongliao, which had substantial populations. Additionally, the current study reflected only the participants seeking healthcare and did not include the community population. A more systematic surveillance is warranted for a comprehensive understanding of brucellosis associated with FUO. Second, further characterization of *Brucella* identified was not successful; therefore, molecular epidemiological analysis was not feasible. Lastly, limited demographic and clinical information was collected, making it infeasible to explore the potential transmission routes beyond animal contact. Although none of the participants were originally diagnosed with brucellosis, the diagnostic test results of the standard serological algorithms for brucellosis could serve as the benchmark for evaluation and interpretation of the qPCR results in the current study, should they be made accessible. Nonetheless, integrating accurate and sensitive *Brucella* testing into the routine diagnostic algorithm for fever is essential for precise treatment, preventing chronic disability and effectively managing the disease across the human–animal interface in Inner Mongolia.

## Data Availability

The original contributions presented in the study are included in the article/**Supplementary Material**. Further inquiries can be directed to the corresponding author.

## References

[B1] Abbas F. Ali S. Muhammad A. Azam A. Moawad A. A. Ejaz M. . (2025). Human brucellosis in the rural and urban population of Pakistan: seroprevalence, risk factors, and clinical manifestations. Curr. Microbiol. 82, 80. doi: 10.1007/s00284-025-04063-x 39797980

[B2] Ali S. Mushtaq A. Hassan L. Syed M. A. Foster J. T. Dadar M. (2024). Molecular epidemiology of brucellosis in Asia: insights from genotyping analyses. Vet. Res. Commun. 48, 3533–3550. doi: 10.1007/s11259-024-10519-5 39230771

[B3] Almas S. Carpenter R. E. Singh A. Rowan C. Tamrakar V. K. Sharma R. (2023). Deciphering microbiota of acute upper respiratory infections: a comparative analysis of PCR and mNGS methods for lower respiratory trafficking potential. Adv. Respir. Med. 91, 49–65. doi: 10.3390/arm91010006 36825940 PMC9952210

[B4] Araj G. F. (2010). Update on laboratory diagnosis of human brucellosis. Int. J. Antimicrob. Agents 36, S12–S17. doi: 10.1016/j.ijantimicag.2010.06.014 20692128

[B5] Becker G. N. Tuon F. F. (2021). Comparative study of IS711 and bcsp31-based polymerase chain reaction (PCR) for the diagnosis of human brucellosis in whole blood and serum samples. J. Microbiol. Methods 183, 106182. doi: 10.1016/j.mimet.2021.106182 33647359

[B6] Cao X. Liu P. Wu J. Liu Z. Zhang Y. Yin C. . (2025). Genome phylogenetic analysis of Brucella melitensis in Northwest China. BMC Microbiol. 25, 208. doi: 10.1186/s12866-025-03943-3 40211148 PMC11983976

[B7] Chen Y. Ke Y. Wang Y. Yuan X. Zhou X. Jiang H. . (2013). Changes of predominant species/biovars and sequence types of Brucella isolates, Inner Mongolia, China. BMC Infect. Dis. 13, 514. doi: 10.1186/1471-2334-13-514 24176041 PMC3819263

[B8] Di Bonaventura G. Angeletti S. Ianni A. Petitti T. Gherardi G. (2021). Microbiological laboratory diagnosis of human brucellosis: an overview. Pathog. (Basel Switzerland) 10, 1623. doi: 10.3390/pathogens10121623 34959578 PMC8709366

[B9] Expert Consensus Group (2017). Expert consensus on the diagnosis and treatment of fever of unknown origin. Chin. J. Infect. Dis. 35, 641–655. doi: 10.3760/cma.j.issn.1000-6680.2017.11.001 30704229

[B10] Fadeel M. A. Hoffmaster A. R. Shi J. Pimentel G. Stoddard R. A. (2011). Comparison of four commercial IgM and IgG ELISA kits for diagnosing brucellosis. J. Med. Microbiol. 60, 1767–1773. doi: 10.1099/jmm.0.033381-0 21835974

[B11] Franco M. P. Mulder M. Gilman R. H. Smits H. L. (2007). Human brucellosis. Lancet Infect. Dis. 7, 775–786. doi: 10.1016/S1473-3099(07)70286-4 18045560

[B12] Gao Z. Jiang Y. Chen M. Wang W. Liu Q. Ma J. (2025). Enhancing fever of unknown origin diagnosis: Machine learning approaches to predict metagenomic next-generation sequencing positivity. Front. Cell. Infect. Microbiol. 15, 1550933. doi: 10.3389/fcimb.2025.1550933 40302920 PMC12037494

[B13] García-Méndez K. B. Hielpos S. M. Soler-Llorens P. F. Arce-Gorvel V. Hale C. Gorvel J. P. . (2019). Infection by Brucella melitensis or Brucella papionis modifies essential physiological functions of human trophoblasts. Cell. Microbiol. 21, e13019. doi: 10.1111/cmi.13019 30817085

[B14] Guo G. Tuohetaerbaike B. Wu X. Zhang Y. Li J. Zhang W. (2023). Serological investigation of brucella infection using a dipstick assay among individuals with unexplained fever in farming-pastoral areas of Xinjiang, China. Diagn. Microbiol. Infect. Dis. 107, 116079. doi: 10.1016/j.diagmicrobio.2023.116079 37827089

[B15] Hanot Mambres D. Boarbi S. Michel P. Bouker N. Escobar-Calle L. Desqueper D. . (2017). Imported human brucellosis in Belgium: Bio and molecular typing of bacterial isolates, 1996-2015. PloS One 12, e0174756. doi: 10.1371/journal.pone.0174756 28384245 PMC5383062

[B16] Hasanjani Roushan M. R. Ebrahimpour S. Moulana Z. (2016). Different clinical presentations of brucellosis. Jundishapur J. Microbiol. 9, e33765. doi: 10.5812/jjm.33765 27284398 PMC4897599

[B17] Hinić V. Brodard I. Thomann A. Cvetnić Z. Makaya P. V. Frey J. . (2008). Novel identification and differentiation of Brucella melitensis, B. abortus, B. suis, B. ovis, B. canis, and B. neotomae suitable for both conventional and real-time PCR systems. J. Microbiol. Methods 75, 375–378. doi: 10.1016/j.mimet.2008.07.002 18675856

[B18] Klietmann W. F. Ruoff K. L. (2001). Bioterrorism: implications for the clinical microbiologist. Clin. Microbiol. Rev. 14, 364–381. doi: 10.1128/CMR.14.2.364-381.2001 11292643 PMC88979

[B19] Lavigne J. P. Magnan C. Loubet P. Sotto A. O'Callaghan D. Keriel A. (2025). What's new in the diagnosis and treatment of human brucellosis? Infect. Dis. Now 55, 105121. doi: 10.1016/j.idnow.2025.105121 40712782

[B20] Lim M. L. Rickman L. S. (2004). Brucellosis. Infect. Dis. Clin. Pract. 12, 7–14. doi: 10.1097/01.idc.0000104894.16995.c4 33079766

[B21] Liu S. Hu J. Zhao Y. Wang X. Wang X. (2025a). Prediction and control for the transmission of brucellosis in inner Mongolia, China. Sci. Rep. 15, 3532. doi: 10.1038/s41598-025-87959-9 39875488 PMC11775140

[B22] Liu S. Hu J. Zhao Y. Wang X. Wang X. (2025b). Prediction and control for the transmission of brucellosis in inner Mongolia, China. Sci. Rep. 15, 3532. doi: 10.1038/s41598-025-87959-9 39875488 PMC11775140

[B23] Liu J. Y. Jiang H. (2021). Application and thinking of diagnostic methods of brucellosis in China. Zhonghua liu xing bing xue za zhi = Zhonghua liuxingbingxue zazhi 42, 160–163. doi: 10.3760/cma.j.cn112338-20200516-00734 33503714

[B24] Liu J. Song Z. Ta N. Tian G. Yang X. Zhao H. . (2023). Development and evaluation of a droplet digital PCR assay to detect Brucella in human whole blood. PloS Negl. Trop. Dis. 17, e0011367. doi: 10.1371/journal.pntd.0011367 37267228 PMC10237378

[B25] Liu S. Soontornchai S. Bovornkitti S. Wang X. (2024). Epidemiological characteristics and spatio-temporal clusters of human brucellosis in Inner Mongolia, 2010-2021. BMC Infect. Dis. 24, 1321. doi: 10.1186/s12879-024-10165-x 39567873 PMC11577583

[B26] Liu Z. G. Wang M. Ta N. Fang M. G. Mi J. C. Yu R. P. . (2020). Seroprevalence of human brucellosis and molecular characteristics of Brucella strains in Inner Mongolia Autonomous region of China, from 2012 to 2016. Emerg. Microbes Infect. 9, 263–274. doi: 10.1080/22221751.2020.1720528 31997725 PMC7034055

[B27] Loubet P. Magnan C. Salipante F. Pastre T. Keriel A. O'Callaghan D. . (2024). Diagnosis of brucellosis: combining tests to improve performance. PloS Negl. Trop. Dis. 18, e0012442. doi: 10.1371/journal.pntd.0012442 39236075 PMC11407618

[B28] Makashir P. Shinde U. Koithara B. Purandare B. (2026). Brucella melitensis sacroiliitis as an unusual culprit for fever of unknown origin (FUO). BMJ Case Rep. 19, e269495. doi: 10.1136/bcr-2025-269495 41526071

[B29] Marks F. Liu J. Soura A. B. Gasmelseed N. Operario D. J. Grundy B. . (2021). Pathogens that cause acute febrile illness among children and adolescents in Burkina Faso, Madagascar, and Sudan. Clin. Infect. Dis. 73, 1338–1345. doi: 10.1093/cid/ciab289 33822011 PMC8528393

[B30] Marra A. R. Lopes G. O. V. Pardo I. Hsieh M. K. Kobayashi T. Marra P. S. . (2024). Metagenomic next-generation sequencing in patients with fever of unknown origin: A comprehensive systematic literature review and meta-analysis. Diagn. Microbiol. Infect. Dis. 110, 116465. doi: 10.1016/j.diagmicrobio.2024.116465 39059148

[B31] Park J. H. Kim T. S. Park H. Kang C. K. (2024). Delay in the diagnosis of Brucella abortus bacteremia in a nonendemic country: a case report. BMC Infect. Dis. 24, 489. doi: 10.1186/s12879-024-09377-y 38741035 PMC11089730

[B32] Patel J. (2026). One Health approach in human brucellosis: a comprehensive review of epidemiology, transmission, diagnosis, management, and control strategies. OHMI 2, 22–35. doi: 10.66585/ohmi.2026.2.0013

[B33] Schelling E. Diguimbaye C. Daoud S. Nicolet J. Boerlin P. Tanner M. . (2003). Brucellosis and Q-fever seroprevalences of nomadic pastoralists and their livestock in Chad. Prev. Vet. Med. 61, 279–293. doi: 10.1016/j.prevetmed.2003.08.004 14623412

[B34] Sha H. Duan Q. Lyu D. Qian F. Zheng X. Guo J. . (2025). Follow-up of antibody changes in brucellosis patients in Gansu, China. Microbiol. Spectr. 13, e0286224. doi: 10.1128/spectrum.02862-24 40304471 PMC12131799

[B35] Shi Q. N. Qin H. J. Lu Q. S. Li S. Tao Z. F. Fan M. G. . (2024). Incidence and warning signs for complications of human brucellosis: a multi-center observational study from China. Infect. Dis. Poverty 13, 18. doi: 10.1186/s40249-024-01186-4 38374211 PMC10877768

[B36] Soares C. N. da Silva M. T. T. Lima M. A. (2023). Neurobrucellosis. Curr. Opin. Infect. Dis. 36, 192–197. doi: 10.1097/QCO.0000000000000920 37093043

[B37] Torres Montaguth O. E. Buddle S. Morfopoulou S. Breuer J. (2026). Clinical metagenomics for diagnosis and surveillance of viral pathogens. Nat. Rev. Microbiol. 24, 61–75. doi: 10.1038/s41579-025-01223-5 40804538

[B38] Vrioni G. Pappas G. Priavali E. Gartzonika C. Levidiotou S. (2008). An eternal microbe: Brucella DNA load persists for years after clinical cure. Clin. Infect. Dis.: An. Off. Publ. Infect. Dis. Soc. America 46, e131–e136. doi: 10.1086/588482 18462106

[B39] Wang X. Tian X. Li W. Yang Y. Zhang S. Wang H. . (2025). An SNP-based diagnostic method for Brucella S2 vaccine strain infections. Front. Vet. Sci. 12, 1570220. doi: 10.3389/fvets.2025.1570220 40612150 PMC12221913

[B40] Wang M. Zheng C. Liu Z. Li Z. (2025). Spatiotemporal distribution of human brucellosis in Inner Mongolia Autonomous Region, 2004–2023. Dis. Surveill. 40, 358–364. doi: 10.3784/jbjc.202407190423

[B41] Wolf J. Goggin K. P. Inaba Y. Allison K. J. Ahmed A. A. Maron G. . (2026). Predicting bloodstream infection by plasma cell-free metagenomic sequencing: a prospective cohort study. Lancet Microbe 7, 101312. doi: 10.1016/j.lanmic.2025.101312 41780551 PMC13154368

[B42] Wright W. F. Simner P. J. Carroll K. C. Auwaerter P. G. (2022). Progress report: next-generation sequencing, multiplex polymerase chain reaction, and broad-range molecular assays as diagnostic tools for fever of unknown origin investigations in adults. Clin. Infect. Dis. 74, 924–932. doi: 10.1093/cid/ciab155 33606012

[B43] Wu Z. G. Song Z. Y. Wang W. X. Xi W. N. Jin D. Ai M. X. . (2022). Human brucellosis and fever of unknown origin. BMC Infect. Dis. 22, 868. doi: 10.1186/s12879-022-07872-8 36411430 PMC9680120

[B44] Xiao Y. Li M. Guo X. Zeng H. Shuai X. Guo J. . (2022). Inflammatory mechanism of Brucella infection in placental trophoblast cells. Int. J. Mol. Sci. 23, 13417. doi: 10.3390/ijms232113417 36362199 PMC9657658

[B45] Xu N. Qu C. Sai L. Wen S. Yang L. Wang S. . (2023). Evaluating the efficacy of serological testing of clinical specimens collected from patients with suspected brucellosis. PloS Negl. Trop. Dis. 17, e0011131. doi: 10.1371/journal.pntd.0011131 36802393 PMC9942959

[B46] Yan J. F. Zhou H. Y. Luo S. F. Wang X. Yu J. D. (2021). Rare case of brucellosis misdiagnosed as prostate carcinoma with lumbar vertebra metastasis: a case report. World J. Clin. cases 9, 6009–6016. doi: 10.12998/wjcc.v9.i21.6009 34368321 PMC8316935

[B47] Yuan B. Jiang T. Han J. Wang M. (2025). Neurobrucellosis with negative serological examination: a case report and literature review. Front. Med. 12, 1583891. doi: 10.3389/fmed.2025.1583891 40547922 PMC12179128

[B48] Zhai J. Peng R. Wang Y. Lu Y. Yi H. Liu J. . (2021). Factors associated with diagnostic delays in human brucellosis in Tongliao City, Inner Mongolia Autonomous Region, China. Front. Public Health 9, 648054. doi: 10.3389/fpubh.2021.648054 34692615 PMC8526552

[B49] Zhang M. Chen X. Bu Q. Tan B. Yang T. Qing L. . (2024). Spatiotemporal dynamics and influencing factors of human brucellosis in Mainland China from 2005-2021. BMC Infect. Dis. 24, 76. doi: 10.1186/s12879-023-08858-w 38212685 PMC10785479

[B50] Zhang J. H. Feng Z. J. Jiang M. Li X. S. Ma J. Q. (2011). Using exploratory spatial data analysis (ESDA) on the regional distribution of human brucellosis in six provinces of north China: 2004-2007. Chin. J. Epidemiol. 32, 1278–1284. 22336617

[B51] Zhang Y. Hai Y. Teng Z. Xie X. Xiong Y. Shao Z. . (2022). Investigation of 9 pathogenic intracellular bacteria in blood samples from outpatients with undefined febrile illness. Dis. Surveill. 37, 457–463. doi: 10.3784/jbjc.202201200010

[B52] Zheng R. Xie S. Lu X. Sun L. Zhou Y. Zhang Y. . (2018). A systematic review and meta-analysis of epidemiology and clinical manifestations of human brucellosis in China. BioMed. Res. Int. 2018, 5712920. doi: 10.1155/2018/5712920 29850535 PMC5937618

