# Supplementary material for: Prevalence and detection of Brucella infection in people with fever of unknown origin in Inner Mongolia, China
Source: Front Cell Infect Microbiol. 2026 Jun 17;16:1838461. doi: 10.3389/fcimb.2026.1838461 (PMC13318869; doi:10.3389/fcimb.2026.1838461)
Supplement: Supplementary file 1 [file Table1.docx]

Supplemental Table S1. Primer and probe sequences of the PCR assays used in this study.

|  | **Target** | **PCR type** | **Gene target** | **Sequences** | **Amplicon length (bp)** |
| --- | --- | --- | --- | --- | --- |
|  | *Brucella* spp. | qPCR | *IS711* | F: GCTTGAAGCTTGCGGACAGT | 63 |
|  |  |  |  | R: GGCCTACCGCTGCGAAT |  |
|  |  |  |  | P: AAGCCAACACCCGGCCATTATGGT |  |
|  | *B. melitensis* | Nested PCR | BMEL1162 | 1^st^ round F:CCTTGATGACTTTCGGCAAA | 856 |
|  |  |  |  | 1^st^ round R:TATCCGGCTTAGAGGGTGTG |  |
|  |  |  |  | 2nd round F:AAATCGCGTCCTTGCTGGTCTGA | 731 |
|  |  |  |  | 2nd round R:TGCCGATCACTTAAGGGCCTTCAT |  |
|  | *B. abortus* | Nested PCR | Alkb | 1st round F:GACGAACGGAATTTTTCCAATCCC | 517 |
|  |  |  |  | 1st round R:GTCATTGCTGATGCAGCCTA |  |
|  |  |  |  | 2^nd^ round F:GACGAACGGAATTTTTCCAATCCC | 489 |
|  |  |  |  | 2^nd^ round R:TGCCGATCACTTAAGGGCCTTCAT |  |


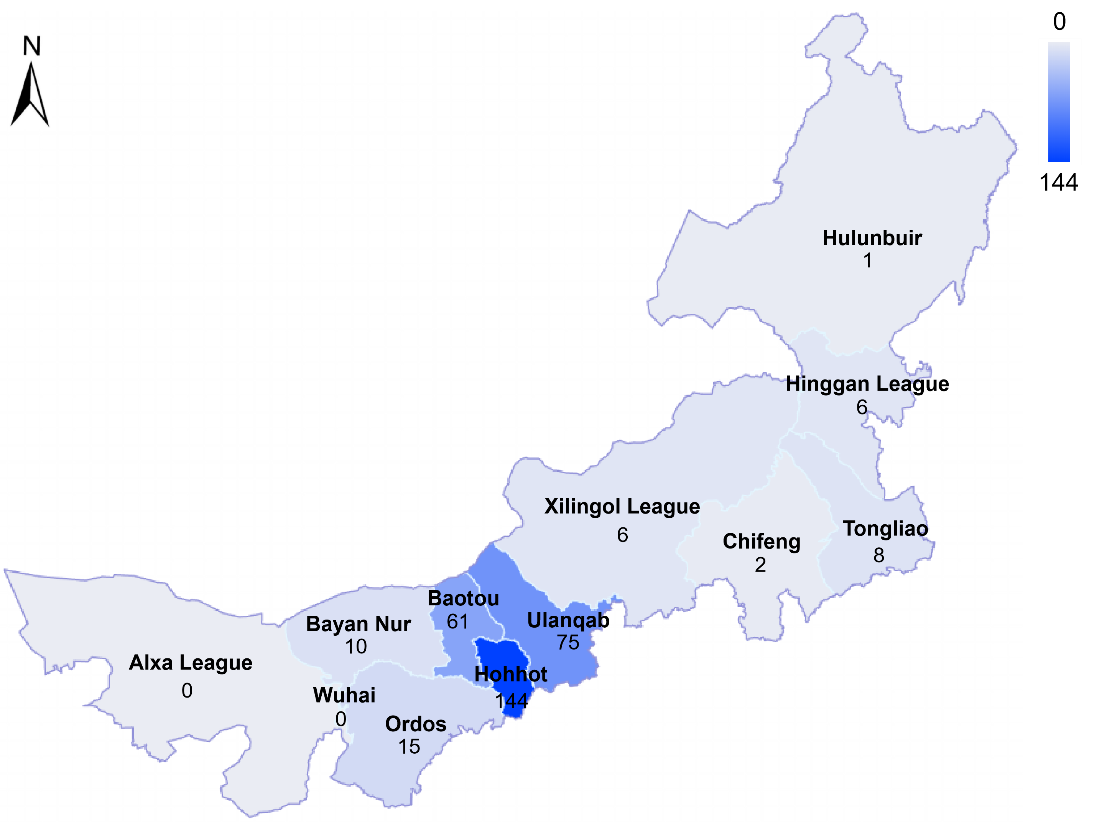


Figure S1 The geographical distribution of the participants in Inner Mongolia in the current study.

Figure S2 Correlation between qPCR Cq values and dPCR copy numbers (log, 10^3^ per mL of blood).

Figure S3 Comparison of Brucella DNA loads for different demographic and clinical features of FUO patients.
